# Oxygen-induced impairment in arterial function is corrected by slow breathing in patients with type 1 diabetes

**DOI:** 10.1038/s41598-017-04947-4

**Published:** 2017-07-20

**Authors:** Luciano Bernardi, Daniel Gordin, Marco Bordino, Milla Rosengård-Bärlund, Anna Sandelin, Carol Forsblom, Per-Henrik Groop

**Affiliations:** 1 Folkhälsan Institute of Genetics, Folkhälsan Research Center, University of Helsinki, Helsinki, Finland; 2 Abdominal Center Nephrology, University of Helsinki and Helsinki University Central Hospital, Helsinki, Finland; 3 Research Program Unit, Diabetes and Obesity, University of Helsinki, Helsinki, Finland; 4 The Baker IDI Heart and Diabetes Institute, Melbourne, Victoria Australia

## Abstract

Hyperoxia and slow breathing acutely improve autonomic function in type-1 diabetes. However, their effects on arterial function may reveal different mechanisms, perhaps potentially useful. To test the effects of oxygen and slow breathing we measured arterial function (augmentation index, pulse wave velocity), baroreflex sensitivity (BRS) and oxygen saturation (SAT), during spontaneous and slow breathing (6 breaths/min), in normoxia and hyperoxia (5 L/min oxygen) in 91 type-1 diabetic and 40 age-matched control participants. During normoxic spontaneous breathing diabetic subjects had lower BRS and SAT, and worse arterial function. Hyperoxia and slow breathing increased BRS and SAT. Hyperoxia increased blood pressure and worsened arterial function. Slow breathing improved arterial function and diastolic blood pressure. Combined administration prevented the hyperoxia-induced arterial pressure and function worsening. Control subjects showed a similar pattern, but with lesser or no statistical significance. Oxygen-driven autonomic improvement could depend on transient arterial stiffening and hypertension (well-known irritative effect of free-radicals on endothelium), inducing reflex increase in BRS. Slow breathing-induced improvement in BRS may result from improved SAT, reduced sympathetic activity and improved vascular function, and/or parasympathetic-driven antioxidant effect. Lower oxidative stress could explain blunted effects in controls. Slow breathing could be a simple beneficial intervention in diabetes.

## Introduction

Diabetes is associated with increased premature cardiovascular morbidity and mortality^1^. Although several factors contribute to these adverse outcomes, autonomic and vascular abnormalities have an adverse prognostic effect both in experimental and epidemiological studies^1^. These abnormalities have traditionally been attributed to anatomical changes involving neural degeneration^2^ or atherosclerotic processes^3^. However, a functional –hence potentially reversible- component of autonomic and vascular abnormalities have been identified by our group, and others^4, 5^.

Following these concepts, we observed that the parasympathetic cardiac arm of the baroreflex could be transiently ameliorated by two simple physiological interventions, namely slow breathing^5^ and oxygen administration^6^. Nevertheless, in response to oxygen administration we also observed a transient increase in the blood pressure in patients with type 1 diabetes^4, 7^. Conversely, slow breathing did not increase or it even reduced the blood pressure^8^. These findings lead to the question of whether these two interventions, which at first sight produce similar autonomic effects, might in fact have entirely different mechanisms of action, based on the responses in blood pressure and arterial function, and its possible consequences in stimulating the parasympathetic nervous system. However, so far, the effects of slow breathing and oxygen on arterial function have never been compared, neither in healthy nor in patients with type 1 (or type 2) diabetes. The question is however relevant in order to understand how the autonomic function is altered in diabetes and how this could potentially be corrected.

We therefore tested whether slow breathing and oxygen administration exhibit different effects on arterial and autonomic function and whether the combined effects of these two interventions further potentiate the vascular or the autonomic response. We then measured the augmentation index and the pulse wave velocity as indices of arterial function, and the baroreflex sensitivity, as a comprehensive index of autonomic function, in response to oxygen and slow breathing during separate and combined administration. We reasoned that if oxygen and slow breathing act through independent mechanisms their combination should produce some additive or even multiplicative effects, whereas if they act through the same mechanism although in opposite directions, their combination should cancel their individual effects.

## Methods

### Participants

We studied 91 patients with type 1 diabetes and 40 age-matched healthy controls. The participants were recruited through the register of The Social Insurance Institution that comprises all patients entitled to special reimbursement of insulin or anti-diabetic medication in Finland. Selection criteria were diabetes (E10 in ICD-10) diagnosed before the age of 35 years, and age 18–35 years at the time of inclusion.

Type 1 diabetes was defined as C-peptide deficiency (<0.03 nmol/l) and initiation of permanent insulin treatment within one year after the diagnosis of diabetes. None of the patients showed clinical signs of cardiovascular disease. Six patients were laser-treated because of diabetic retinopathy.

Nineteen patients received antihypertensive medication (14 with an ACE-inhibitor, 1 with a combination of an ACE-inhibitor, a calcium channel blocker and a diuretic, 1 with a combination of ACE-inhibitor and calcium channel blocker, and 3 with angiotensin-2 receptor blockers). The healthy control participants were recruited by email advertisements among university students and staff. Only individuals with normal fasting glucose and without 1^st^ degree relatives with diabetes mellitus were included. Before participation, all participants gave their written informed consent. The study protocol was approved by the Ethics Committee of Helsinki University Hospital, and the study was carried out in accordance with the principles of the Declaration of Helsinki as revised in 2000.

The participants underwent a clinical examination, resting-ECG, laboratory testing, overnight urine collections, and standard autonomic function evaluation by 4 cardiovascular tests: the expiration/inspiration ratio of the RR interval during slow deep breathing, the maximum/minimum 30/15 ratio of the RR interval during Valsalva manoeuvre and active standing, the systolic blood pressure response to standing. Cardiovascular autonomic neuropathy was defined as the presence of two or more abnormal tests^9^. Each patient completed a detailed questionnaire on life style, smoking habits and family history. Anthropometric data of the participants are shown in Table 1.

**Table 1:** Clinical characteristics and laboratory measurements of patients with type 1 diabetes and healthy control subjects.

	Type 1 diabetes	Controls	*p-*value
N	91	40	
Gender (men/women)	50/41	19/21	NS
Age (years)	31.5 ± 0.6	31.0 ± 1.1	NS
Duration of diabetes (years)	13.2 ± 0.2	0	
Age at onset (years)	18.0 ± 0.6	—	
Body Mass Index (kg/m2)	25.7 ± 0.4	23.8 ± 0.6	<0.05
Waist/Hip ratio	0.87 ± 0.1	0.86 ± 0.01	NS
Current smokers (n,%)	14, 15.4%	5, 12.6%	
Antihypertensive treatment (n,%)	19, 20.8%	0	
Laser-treated retinopathy (n,%)	6, 6.2%	0	
Microalbuminuria (n,%)	6, 6.6%	0	
Macroalbuminuria (n,%)	1, 1%	0	
HbA1c, % (mmol/mol)	8.03 ± 0.12 64.4 ± 1.3	5.25 ± 0.04* 33.7 ± 0.5*	<0.001
Total cholesterol (mmol/l)	4.58 ± 0.08	4.35 ± 0.15*	NS
HDL-cholesterol (mmol/l)	1.63 ± 0.05	1.62 ± 0.09*	NS
Triacylglycerol (mmol/l)	1.23 ± 0.08	0.85 ± 0.07*	<0.05
Urinary AER (mg/24 h)	23.8 ± 11.7	6.72 ± 1.10*	NS
Serum creatinine (μmol/l)	68.7 ± 1.2	72.2 ± 2.7*	NS
Estimated Glomerular Filtration Rate (ml min^−1^ 1.73 m^−2^)	167 ± 6	148 ± 8	NS
Office SBP (mmHg)	129.8 ± 1.2	120.9 ± 2.7	<0.005
Office DBP (mmHg)	77.2 ± 0.9	75.1 ± 1.4	NS
Autonomic score	0.20 ± 0.04	0.12 ± 0.05	NS

SBP: Systolic Blood Pressure. DBP: Diastolic Blood pressure* data obtained in 24 control subjects.

### Protocol

All participants were investigated in a quiet room, at a temperature between 19 and 23 °C, between 8 a.m. and 2 p.m. The participants received instructions to refrain from alcohol for 36 h, caffeinated beverages and cigarettes for 12 h prior to the examination. A light meal was permitted 2 hours before testing. If a participant reported or measured symptoms/values of hypoglycaemia in the previous 24 hours the test was postponed. The electrocardiogram was recorded using a bipolar precordial lead. Continuous blood pressure was monitored with a Finapres 2300 digital plethysmograph (Ohmeda, Louisville, CO, USA). Two respiratory signals were obtained by inductive plethysmography (Z-rip®, Pro-Tech, Mukilteo, Wa, USA), from belts positioned around the chest and the abdomen. Pulse oxymetry and expired carbon dioxide (CO_2_) partial pressure (7100 model CO2SMO, Novametrix, Wallingford, CT, USA) were obtained.

The signals were recorded in the supine position during 5 minutes of spontaneous breathing, and during 2 minutes of slow deep breathing at the rate of 6 cycles/minute. Subsequently, the participants repeated the entire protocol while breathing 5 L/min oxygen. Signal recordings started after the first 5 minutes of oxygen administration to allow stabilization of oxygen saturation and ventilation. All signals were simultaneously recorded on a personal computer with an analog-to-digital converter with a 12-bit resolution at a sampling rate of 200 Hz (WinAcq data acquisition system, Absolute Aliens Ltd, Turku, Finland).

### Assessment of baroreflex sensitivity

From the original data, the time series of the RR interval (from each of 2 consecutive R waves of the electrocardiogram) and the systolic blood pressure (SBP) were obtained. Previous studies have shown poor correlation between different indices of BRS, while no method has shown superior performance over the other^10^. Accordingly, we computed all 7 most common BRS indices, and used their average^11^.

BRS was determined from spontaneous fluctuations in the RR interval and SBP during the spontaneous and 6 breaths/min recordings using the sequence methods for (1) positive and (2) negative sequences, or spectral analysis for the (3) low frequency, (4) high frequency and (5) for the average of the low- and high-frequency components, (6) the transfer function technique and (7) by the standard deviation method, following the technical details previously explained^11^. Additionally, the standard deviation of all RR intervals (SDNN) was considered an index of global RR interval variability.

### Assessment of arterial stiffness

Using the ECG R-wave peak as a reference, we obtained an average pulse pressure profile during each recording. From this averaged pressure signal we obtained the augmentation index, adjusted for the standard heart rate of 75 beats/min (AI75) using a recently validated method^12^. The pulse wave velocity (PWV) was calculated as the equivalent index SI-DVP derived from the pulse pressure profile, described by Millasseau *et al*. and validated by themselves^13^ and by our group^12^.

### Laboratory tests

Venous blood samples were obtained after a light breakfast and were analyzed for HbA_1c_, lipids and serum creatinine. HbA_1c_ concentrations were determined by an immunoturbidimetric immunoassay (Medix Biochemica, Kauniainen, Finland). Serum lipids (cholesterol, triglycerides, HDL-cholesterol) and creatinine were measured by enzymatic methods. Urinary albumin excretion rate (AER) was measured from three consecutive timed urine collections, one 24-hour and two overnight collections. Normal AER was defined as values persistently < 20 μg/min or < 30 mg/24 h, microalbuminuria as AER ≥ 20 < 200 µg/min or ≥ 30 < 300 mg/24 h, and macroalbuminuria as AER ≥ 200 µg/min or ≥ 300 mg/24 h in at least two out of three urine collections^5^.

### Statistical analyses

Data are presented as mean ± standard error of the mean, unless differently stated. Differences between the two groups and between conditions were tested by a linear normal model. As outcome we modeled the different continuous variables, and included conditions (normoxia/hyperoxia), breathing patterns (spontaneous, and slow breathing) and participant groups (healthy control/diabetic) as categorical covariates. For each continuous variable, we assessed the interactions between conditions, groups and breathing patterns. Sheffe’s test was used to test for significances between different breathing rates. Statistical significance was defined as a p-value ≤ 0.05.

## Results

### Baseline data

**Figure 1:**
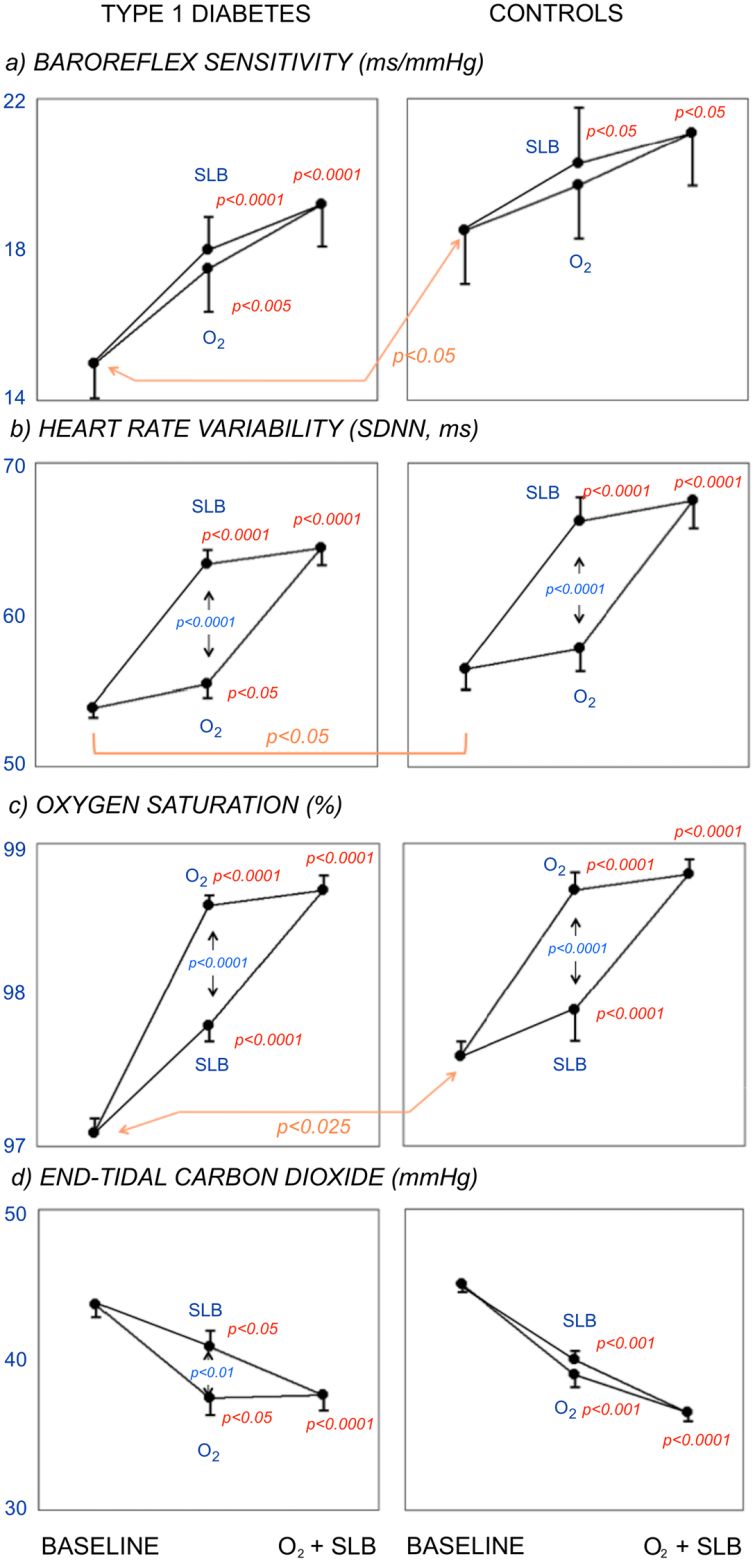
Effect of Slow Breathing (SLB) and Oxygen (O_2_), alone (middle data of each panel) and combined (right data in each panel) on autonomic function: baroreflex sensitivity (panels a) and heart rate variability (standard deviation of RR intervals, SDNN, panels b), and blood gases: oxygen saturation (panels c) and end-tidal carbon dioxide (CO_2-et_, panels d). Data from Type 1 diabetic (left panels) and control participants (right panels). Baseline: spontaneous breathing in room air. Within each panel, significances written in red refer to baseline, significances in blue refer to SLB vs O_2_. Note the additive effect of oxygen and slow breathing in all autonomic and respiratory variables.

**Figure 2:**
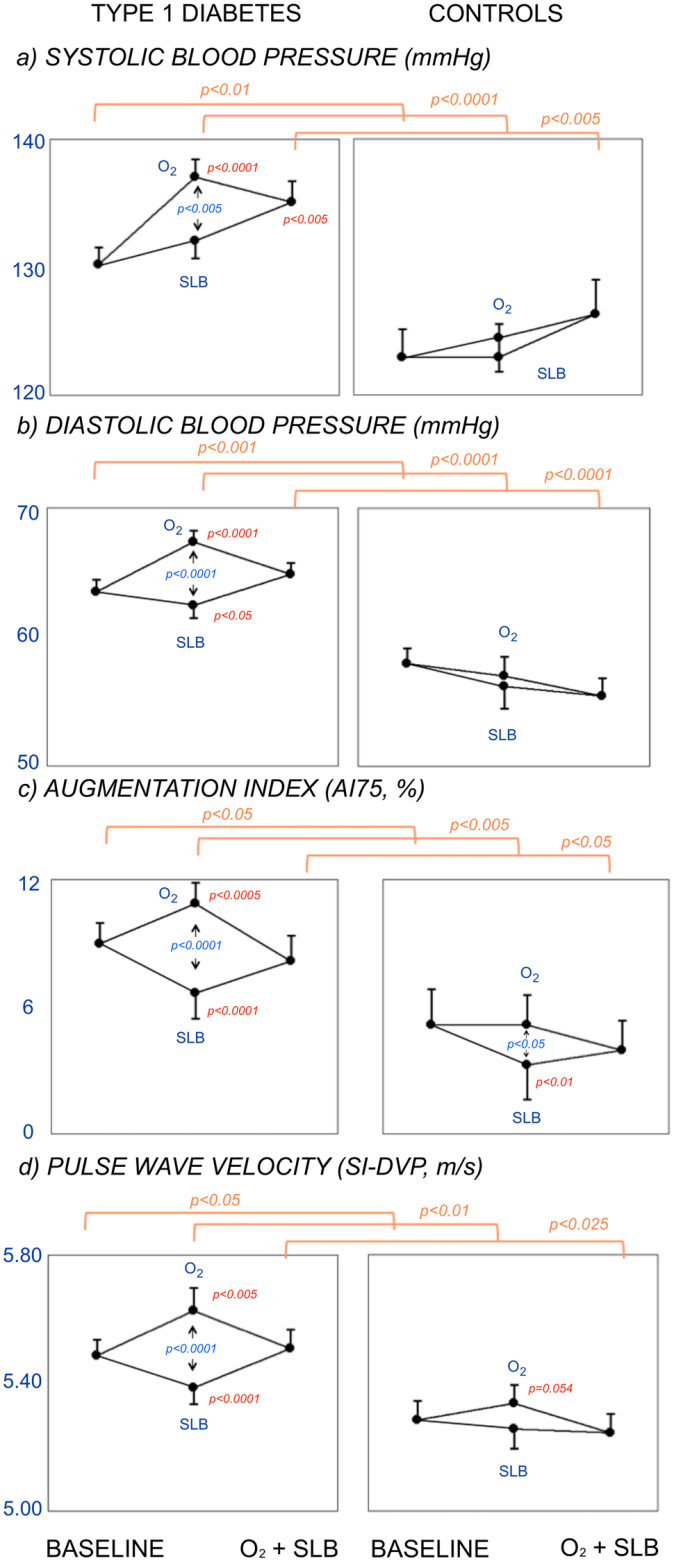
Effect of Slow Breathing (SLB) and Oxygen (O_2_), alone (middle data of each panel) and combined (right data in each panel) on blood pressures: systolic (panels a) and diastolic (panels b), and arterial function: heart rate-adjusted augmentation index (AI75, panels c) and Pulse wave velocity (SI-DVP, panels d). Same explanations as in Fig. 1. Note the opposite effects of slow breathing and oxygen on blood pressure and particularly on each index of arterial function, and the blockade of the oxygen-induced worsening by combining slow breathing.

During spontaneous breathing in normoxia, diabetic subjects had lower BRS and SDNN (Fig. 1, panels a and b), lower resting oxygen saturation (Fig. 1, panel c), higher systolic and diastolic blood pressure (Fig. 2, panels a and b), and higher AI75 and PVW than controls (Fig. 2, panels c and d). Mean RR interval was similar in diabetic and control subjects (940 ± 15 vs 1016 ± 17 msec, NS). No differences were observed in the end-tidal carbon dioxide.

### Effect of oxygen administration

Oxygen administration prolonged the RR interval in diabetic patients (from 940 ± 15 to 1016 ± 17 msec, p < 0.001) and in control subjects (from 971 ± 27 to 1029 ± 25 msec, p < 0.001), and increased BRS, SDNN and oxygen saturation in diabetic and control participants (Fig. 1, panels a, b and c). However, oxygen increased the blood pressure (Fig. 2, panels a and b) and worsened the arterial function (both AI75 and SI-DVP), and this was particularly seen in the diabetic participants (Fig. 2, panels c and d). Control subjects showed similar directional changes but to a lesser degree than in the diabetic participants (Figs 1 and 2). Carbon dioxide levels dropped in both groups (Fig. 1, panel d), indicating some degree of hyperventilation.

### Effect of slow breathing

Similar to oxygen, slow breathing increased BRS and SDNN (Fig. 1, panels a and b) in the diabetic participants, and showed a trend toward an increase in BRS and SDNN in the control participants, whereas the RR interval slightly shortened in the diabetic (to 927 ± 13 msec, p < 0.01) and the control participants (to 946 ± 23 msec, p < 0.025). The extent of the increase in BRS was similar to that of oxygen and it was larger in diabetic (p < 0.0001) than in control (p < 0.05) participants (Fig. 1, panel a). Oxygen saturation also increased in diabetic and control participants (p < 0.001) though less than with oxygen. Unlike oxygen, slow breathing did not increase the systolic blood pressure and decreased the diastolic blood pressure (Fig. 2, panels a and b), and improved the arterial function in the diabetic participants (both AI75 and pulse wave velocity, Fig. 2, panels c and d). In the control group the changes were directionally the same, but a significant improvement was seen only in the augmentation index. Carbon dioxide levels dropped significantly in both groups (Fig. 1, panel d), although to a lesser extent than with oxygen.

### Effect of combined oxygen administration and slow breathing

The combination of oxygen administration and slow breathing did not further increase BRS and SDNN as compared to each intervention separately (Fig. 1, panels a and b), and the RR interval returned to values similar to those at baseline (diabetic group: 955 ± 14 msec, NS; control group: 986 ± 20 msec, NS). Oxygen saturation did not increase further as compared to each separate intervention (Fig. 1, panel c). However, the worsening of the arterial function and the increase in the blood pressures observed with oxygen alone were abolished by adding slow breathing (Fig. 2). Similarly, the worsening of the arterial function (both AI75 and pulse wave velocity) seen with oxygen disappeared when slow breathing was added to oxygen (Fig. 2, panels c and d). Carbon dioxide levels remained lower in both groups (Fig. 1, panel d).

## Discussion

There are several important observations in this study. First, although oxygen and slow breathing produce similar favorable autonomic modifications, their effects are the opposite with respect to blood pressure and particularly arterial function: oxygen impaired and slow breathing improved them. Second, during their combined administration the increase in blood pressure and the impaired arterial function seen with oxygen alone was reversed by slow breathing.

One likely mechanism of the oxygen-induced vasoconstriction and the arterial function worsening is free radical-induced transient endothelial dysfunction. Reactive oxygen species (ROS) induced by excess oxygen are known to have an irritative effect on the bronchial receptors, which could stimulate the parasympathetic activity^14^. Finally, an increase in the blood pressure stimulates the baroreflex as a compensatory mechanism. This hypothesis is supported by the blunted overall effects seen in the healthy controls, as their likely lower baseline oxidative stress could have reduced the impact of oxygen-induced ROS.

Conversely, slow breathing in room air improved blood oxygenation by better ventilation-perfusion matching^15^, and improved the autonomic and arterial function by reducing sympathetic activation at the central and the peripheral levels. The reversal of the impaired arterial function caused by slow breathing during oxygen administration suggests that slow breathing could have a protective effect both on the autonomic and the arterial function, possibly by preventing the negative effects of oxygen-induced ROS on endothelial function, thus “*de facto*” providing an endogenous anti-oxidant effect. This new finding might have important clinical implications.

### Is oxygen administration always beneficial for the patient?

The expected increase in oxygen saturation induced by oxygen was accompanied by a marked increase in the RR interval (decrease in heart rate) and by an improvement in the baroreflex sensitivity. However, these positive findings were accompanied by an increase in the systolic and the diastolic blood pressures, and by an impairment of the arterial function (indicated by an increase in the augmentation index and the pulse wave velocity index). Since resting oxygen saturation was lower in the diabetic as compared to the control group, it is possible that these positive effects on the autonomic function could be due to correction of pre-existing hypoxia. In fact, hypoxia is a direct arterial vasodilator, and as a consequence of the resulting hypotension it is also a stimulator of the sympathetic nervous system. Additionally, hypoxia stimulates the sympathetic nervous system^16^. Accordingly, oxygen-induced relief of the hypoxia, could have induced a vasoconstriction and a simultaneous decrease in the sympathetic activity, and an increase in the parasympathetic activity.

Another mechanism is also plausible and not in contrast to the first. Because an excess of oxygen produces an excess of ROS, both of these might in concert have created a transient endothelial dysfunction, leading to an increase in arterial stiffness and blood pressure^4, 7^. Diabetic subjects already have a condition of oxidative stress, excess of free-radicals and endothelial dysfunction due to various reasons, including hyperglycaemia, low-grade inflammation^17^ and pre-existing hypoxia^6^. These conditions taken together might lead to a low antioxidant reserve, and thus the negative effects of oxygen may be much more pronounced in the diabetic than in the healthy subjects. In our control participants a sufficient antioxidant reserve could have limited the negative effects of oxygen. If this was the case, then “excess” oxygen in the lungs could have initiated an irritative response, already started by receptors present in the lungs^14^.

### Does slow breathing mimic the effects of oxygen?

Slow breathing increases oxygen saturation in healthy subjects^6^, in subjects with heart failure^15^ and diabetes^6^. This effect is due to a reduction in both the anatomic (due to reduced breathing rate) and the physiological dead space (due to improvement in ventilation/perfusion matching). Additionally, slow breathing improves the parasympathetic arm of the cardiac baroreflex in healthy subjects^5^, as well as in diabetic^5^ and cardiac^18^ patients, and directly reduces the muscle sympathetic nerve activity^19, 20^. Because oxygen saturation was depressed in our diabetic participants at baseline, it is likely that some of the effects of slow breathing could be mediated at least in part by improving the arterial oxygenation.

Unlike oxygen, slow breathing reduced the arterial pressure in subjects with essential hypertension^8^, and in the present study, we observed a significant decrease in the diastolic blood pressure. The lack of significant decrease in the systolic blood pressure could be explained both by an overall lower blood pressure in our diabetic patients as compared to the hypertensive subjects^8^, and by the fact that a substantial proportion of them were receiving antihypertensive medication.

Unlike oxygen, slow breathing improved the arterial function (both PWV and AIx75) in our diabetic patients. The improvement in arterial function could simply be a reduction in the sympathetic activity, but this seems to contrast with the fact that also oxygen administration reduced the sympathetic activity, and yet induced an opposite effect on the arterial function.

### Does slow breathing act as an anti-oxidant in type 1 diabetes?

Assuming that the adverse effects of oxygen on arterial function were due to transient endothelial dysfunction induced by excess of free-radicals^7^, then our results lead to the hypothesis that slow breathing may exert some antioxidant effect, possibly via parasympathetic stimulation. The concept that parasympathetic stimulation has an antioxidant effect is well known and supported by many findings in experimental^21–25^ and clinical cardiovascular research, related to heart failure^21, 26, 27^ and stroke^28, 29^.

Although our findings are in full agreement with these previous studies, it is however of note that our results were obtained by slow breathing, and not by direct vagal nerve stimulation. However, it is well established that slow breathing directly suppresses muscle neural sympathetic activity^19, 20^, and recent papers did show that slow breathing has a direct antioxidant effect in healthy subjects, when their redox status was challenged by postprandial hyperglycemia^30^ or acute physical exercise^31^. Ventilatory manipulations, such as slow deep breathing, have been shown to engage neural substrates that partially overlap those found in studies of vagus nerve stimulation. Within the brain, this overlap occurs particularly in the dorsal anterior cingulate cortex, as well as in the superior frontal gyrus and the temporal pole^32–34^. Furthermore, changes in the tidal volume induced by slow deep breathing correlate positively with the activity of the medullary nuclei, including the solitary tract^33^. The same network has been shown to play an important role in vagus nerve stimulation, as vagal afferents traverse the brainstem in the solitary tract, with terminating synapses located mainly in the nuclei of the dorsal medullary complex of the vagus^32^.

In diabetes, similar studies are surprisingly missing, possibly due to the old but now updated concept that autonomic dysfunction is already the result of a neural lesion rather than a complex reflex imbalance, at least at the earlier stages of the disease^5, 6^. The lack of dedicated studies is surprising given that oxidative stress is a frequent finding in diabetes and an established cause of diabetic microvascular complications^17^.

### “Neuropathy” and vascular abnormalities in diabetes: always irreversible?

This paper underlines for the first time that the increase in arterial pressure and stiffness induced by oxygen administration, could be acutely corrected by a simple maneuver that activates the parasympathetic system, thus confirming that the parasympathetic system is not destroyed but only inhibited at the earlier stages of diabetes, and that the vascular abnormalities are also to some extent functional, hence potentially reversible.

### Hypoxia and diabetes

Diabetic patients have lower resting oxygen saturation. Although the oxygen saturation was only mildly reduced, one should consider that in the normoxic range eve a small difference in oxygen saturation implies a large difference in arterial partial O_2_ pressure, due to the dissociation curve of the hemoglobin. This reduced arterial partial O_2_ pressure (hypoxia) could play a relevant role in diabetes, since hypoxia is known to be another relevant source of endothelial dysfunction and ROS generation^35^. Hypoxia could thus be yet another mechanism leading to endothelial dysfunction and oxidative stress in diabetes. The fact that oxygen saturation is reduced at rest also implies a reduced sensititvity of the mechanisms that regulate hypoxia in diabetes, and this has been confirmed in experimental human and animal models^36, 37^. In the present study, we can speculate that the pre-existing ROS excess (likely induced by hypoxia and other mechanisms) could have been increased by oxygen administration, in view of the limited antioxidant reserve of diabetic patients.

Our group previously reported that acute hyperglycaemia can induce oxidative stress and increase arterial stiffness^38, 39^. The fact that our diabetic participants had rather high HbA1c values (8.03 ± 0.12, Table 1) indeed suggests a condition of oxidative stress. In addition, our present and previous findings suggest that with better glycaemic control the adverse vascular response to hyperoxia (or hypoxia) might be reduced. Conversely, the higher resting oxygen saturation of healthy participants, and the concomitant likely lack excess in ROS can explain the reduced modifications observed in the present study.

In summary, while oxygen administration seems to improve the BRS in response to impaired arterial function (possibly by irritative effect of free-radical excess on endothelial function), slow breathing could improve BRS by improving oxygen saturation and by direct parasympathetic stimulation and sympathetic suppression. The effects of slow breathing are fully consistent with a direct antioxidant effect, similarly to what shown for vagal nerve stimulation in other diseases, in which, like in diabetes, oxidative stress plays a major role. There is indeed evidence that slow breathing has a direct antioxidant effect in healthy subjects^30, 31^. These findings could have not only theoretical but also practical interest for the management of diabetes and its complications. However, further studies need to confirm this concept by a direct measure of ROS generation and antioxidant capacity in diabetes.
